# How perceived substance characteristics affect ethical judgement towards cognitive enhancement

**DOI:** 10.1371/journal.pone.0213619

**Published:** 2019-03-14

**Authors:** Eric Mayor, Maxime Daehne, Renzo Bianchi

**Affiliations:** Institute of Work and Organizational Psychology, Faculty of Sciences, University of Neuchâtel, Neuchâtel, Switzerland; Groupe ESC Dijon Bourgogne, FRANCE

## Abstract

Some individuals seek to enhance their cognitive capabilities through the use of pharmacology. Such behavior entails potential health risks and raises ethical concerns. The aim of this study was to examine whether a precursor of behavior, ethical judgement towards the use of existing biological cognitive enhancers (e.g., coffee, legal and illegal drugs), is shaped by the perceived characteristics of these cognitive enhancers. Students and employees completed an online questionnaire which measured perceived characteristics of 15 substances presented as potential cognitive enhancers and a measure of ethical judgement towards these cognitive enhancers. Results of mixed model regression analyzes show that ethical judgement is more favourable when cognitive enhancers are perceived as being legal, familiar, efficient, and safe for users’ health, supporting all hypotheses. Results further show that 36% of variance (in the null model) lies at the level of cognitive enhancers and 21% at the level of participants. In conclusion, cognitive enhancers vary widely in terms of ethical judgement, which is explained by the perception of the mentioned characteristics. Implications regarding prevention and policy-making are discussed.

## Introduction

Cognitive enhancement (CE) refers to the use of technology to improve cognitive characteristics (e.g., memory, attention) and thereby human performance [1]. As for technological innovation in general, CE is often faced with skepticism [2], but also has a non-negligible fraction of supporters among the public [3] and in the media industry [4]. Determinants of ethical support towards CE remain to be better understood. The present study examines whether ethical judgement towards the use of existing biological cognitive enhancers (edible or drinkable substances, notably) is related to their perceived characteristics.

### Ethical judgement

Ethical judgement refers to normative aspects of attitudes in terms of “ought be done” or “ought not be done” on moral grounds [5]. Ethical aspects of CE were discussed for more than a decade in the literature (e.g., [6]). Experts in the field identified a number of ethical concerns related to CE, such as attested risks to health safety [6, 7], the risk that individuals might be coerced into using CE [6–11], the difficulty regulating the use of CE [6], the reduced authenticity of performance obtained using CE [6–8], which is related to an alleged unfair advantage that CE provides to its users in comparison to non-user [8]—a nuanced perspective on the relevance of the unfairness argument is provided by [12]. Finally, unequal access to CE might further inequality in outcomes [7]. The public shares these concerns [10]. Yet, the characteristics that affect ethical judgement regarding the use of individual cognitive enhancers (e.g., caffeine vs cocaine) remain largely unidentified.

Clarifying the factors which affect ethical judgement towards cognitive enhancers is important because of the impact such judgements can have on the actions of individuals [5, 13, 14], including on the consumption of different cognitive enhancers if any (e.g., [15, 16] but see [17]). This issue has yet been only scarcely investigated. This is relevant in light of the health risks potentially associated with the reliance upon CE (e.g., [18]).

### Substance characteristics

Researchers investigated whether the ethical judgement of students towards CE is affected by characteristics of fictitious cognitive enhancers–risks to health and legal status [19] using the vignette method (see [20]). Our study extends the research carried out by [19] by (a) using participants’ assessments of existing substances, rather than relying on the vignette method. The vignette and survey methods complement each other. While the vignette method allows for experimental control, it lacks ecological validity (the validity of the application of the finding of a study in the real world). The survey method allows the investigator to study participants’ view of existing, rather than hypothetical, cognitive enhancers. As such, the survey method has a higher ecological validity. Indeed, although some studies found convergent findings using vignette the method and real-world assessments (e.g., [21]), doubts have been raised regarding the ecological validity of the vignette method; (b) investigating each characteristic’s unique contribution (above and beyond that of others) to variance in ethical judgement; (c) including working adults in our sample; and (d) examining additional characteristics of cognitive enhancers, selected from the literature.

The additional characteristics that we assessed are participants’ familiarity with the effects of target cognitive enhancers and participants’ perception of the effectiveness of target cognitive enhancers. Other characteristics mentioned in the literature focusing on general concerns about CE (e.g., risk of coercion, fairness, and social equality) were not examined because we did not expect them to bear differently on ethical judgement towards the use of individual substances, which is the focus of this study.

### Hypotheses

Below, we briefly examine why each characteristic should be related to ethical judgement and present our hypotheses.

#### Familiarity

Humans might be hardwired to perform more favourable judgements of targets with increased familiarity [22–24]. Such preference might be generalized, as shown by the diversity of types of targets for which it has been demonstrated (e.g., [25–29]. In the specific case of CE, familiarity was indeed found to be a predictor of favourable ethical judgement [30].

Hypothesis 1: Familiarity with a given cognitive enhancer is positively associated with ethical judgment towards the use of that cognitive enhancer.

#### Effectiveness versus risk

Our hypotheses concerning the favourable ethical judgement of effective cognitive enhancers and the unfavourable ethical judgement of cognitive enhancers assessed as entailing health risks draw on utilitarianism. Utilitarianism posits that behaviour is to be judged by its consequences: the attainment of valued states or goals and the minimization of suffering [31]. Utilitarianism prescribes the manner in which decisions are to be made and judged. Theories of actual decision-making often draw on utilitarianism. For instance, Bernoulli’s theory of moral utility—and its development, expected utility theory—posit that individuals decide to perform behaviour that increases utility (the good consequences) and decreases disutility (the bad consequences) [32]. Similar propositions are made in rational choice theory (people weigh in expected utility and social pressure to determine preference [33]) and prospect theory (under uncertainty, losses outweigh gains [34]). The association of perceived effectiveness (utility) and behavioural preferences has been found to be robust in a variety of domains (e.g., [35, 36]). [12] argues that a cultural focus on results is related to an utilitarian perspective that makes the use of CE appealing. Indeed, the effectiveness of cognitive enhancers is a predictor of their intended use [37]. Favourable ethical judgement might underlie such increased use, as people act upon their normative attitudes.

Similarly, behaviour perceived as risky is generally considered undesirable, notably because it elicits negative emotions (e.g., anxiety) and negative utility appraisals [38]. Indeed, negative emotions lead to unfavourable ethical judgement [39]. The same is true for behaviour perceived as unhealthy. On the contrary, behaviour perceived as healthy leads to favourable ethical judgement (e.g., [40–42]).

Hypothesis 2: Perceived effectiveness of the cognitive enhancer is positively associated with ethical judgment.
Hypothesis 3: Perceived health risk of the cognitive enhancer is negatively associated with ethical judgment.

#### Legality

With obvious exceptions, people have internalized, through socialization, general conventional values that include a moral obligation for all to obey the law ([13, 43]). While some scholars have claimed that the law intrinsically creates the moral duty for citizen to follow its rules (e.g., [44, 45]), others have shown that people notably rely on the law and on its enforcement as an indicator of the values of their community, adjust their ethical judgement of behavior on this basis and in their majority act accordingly [46–50].

Hypothesis 4: Perception of legality of the cognitive enhancer is positively associated with ethical judgment.

## Method

### Participants and procedure

Participants took part in an online survey. They were recruited on the campus of a Swiss university and on the social media. Participant recruitment was performed through two major means: Some participants were recruited through e-mails (convenience sampling), which included a short description of the study, a link to the survey, and a request to forward the email to their contacts. Other participants were recruited through posts on social media, which displayed the same content as the e-mails (linkedin, facebook).”

Among the 197 participants who took part in the study, 148 (68% women; 33% students, 57% employees, 10% other), who answered all items relating to at least one cognitive enhancer, were included in our analyses. The average age of the participants was 30.46 years old (*SD* = 11.06; 13 missing values). The participants were asked to assess 15 individual cognitive enhancers on several characteristics. Examples of cognitive enhancers include: Adderall, Amphetamines, Caffeine (e.g., coffee, tea), Methylphenidate (e.g., Ritalin), Tyrosine. The full list is presented in the Appendix. The cognitive enhancers were selected on the basis of their recurrence in the literature, specialized blogs and internet forums; and, for over-the-counter supplements, availability to the Swiss population.

This study was performed in compliance with the Declaration of Helsinki and the guidelines of the Swiss Psychological Society. Participation was voluntary and no compensation was offered for participating. An informed consent page was included on the first page of the questionnaire. Only participants who gave their informed consent were able to take part in the study. On the informed consent page, participants were informed that the aim of the study was to understand how cognitive enhancement was viewed by the public, that the answers they provided would be treated confidentially and that they could interrupt their participation at any time. For the above mentioned reasons, and because the study entailed no foreseeable risks for participants, in was not deemed necessary to seek approval by an ethics committee.

### Measures

Participants first provided socio-demographic information (e.g., gender, age, occupation). After reading a definition of CE, participants completed, for each cognitive enhancer, several single-item measures we designed. We note that single items assessing general constructs have comparable test-retest reliability to multi-item scales and comparable predictive validity, are economical in terms of participant time and burden, and have therefore been recommended by some authors (e.g., [51–53]). Our predictors were measured for each cognitive enhancer as follows:

#### Familiarity

Familiarity with the cognitive enhancers was assessed with the item “Please indicate your degree of familiarity with the following substances”, scored on a 4 points Likert scale: *1—I have never heard about this substance*, *2—I have heard about this substance but I do not know its effects*, *3—I have heard about this substance and have some knowledge of its effect*, and *4—I have heard about this substance and know a lot about its effects*.

#### Perceived effectiveness

Perceived effectiveness for CE was measured with the item “Do you think these substances are effective to increase performance?” rated on a Likert scale (from 1 = *Not at all effective* to 6 = *Absolutely effective*). The response “I don’t know” was also available.

#### Health risk

Health risk was assessed with the item “Do you think the use of these substances to increase performance entails risks for the health of the users?” (rated from 1 = *Not at all risky* to 6 = *Extremely risky*). The response “I don’t know” was also available.

#### Perception of legality

Legal status was assessed by asking participants to classify each cognitive enhancer in one of 3 categories: “*Illegal*”, “*Legal*, *with a medical prescription*”, “*Legal*, *sold over-the-counter*”. The category “Illegal” was the reference category in our analyses.

#### Ethical judgement

The criterion variable, ethical judgment was measured for each cognitive enhancer using the following item: “Do you think the use of these substances to increase performance is ethical (i.e., in accordance with the values of our society)?” (scored from 1 = *Not at all ethical* to 6 = *Absolutely ethical*). The response “I don’t know” was also available.

#### Data preparation

We restructured the dataset from variables to cases. In other words, each of the 15 cognitive enhancers for each of the participants became one row of data. From these observations, we discarded those that were incomplete (listwise deletion), i.e., observations featuring one or more “I don’t know” answers, or no answers because they could not be included in the analyses. The remaining number of observations was 850, assessed by a total of 148 participants (each assessing on average 5.74 cognitive enhancers, *SD* = 2.27). Most participants were able to fully assess only cognitive enhancers of which they knew the effects (the last two modalities of variable ‘familiarity’ represent 91% of the 850 included observations).

## Results

Zero-order correlations between the perceived characteristics of the cognitive enhancers are provided in Table 1. Ethical judgement correlated significantly with all other studied variables: positively with familiarity, effectiveness and negatively with health risk (strongest correlation with ethical judgement: *r* = .46). The cognitive enhancers that were perceived to be legal received a more favourable ethical judgement than those that were not, and were considered less risky. Finally, effectiveness correlated negatively with health risk.

**Table 1:** Zero-order correlations among the study variables (N = 850).

	*M*	*SD*	1.	2.	3.	4.
1. Familiarity	3.46	.71	—			
2. Effectiveness	2.94	1.66	.029	—		
3. Risk	4.17	1.78	-.087	-.334	—	
4. Legal	.79	.40	.237	-.024	-.449	—
5. Ethical judgement	3.11	2.02	.242	.315	-.634	.462

Correlations above .07 are statistically significant at p < .05. Variable Legal in this table is coded 0 for the legal status “Illegal” and 1 otherwise.

Data were analysed using random intercepts mixed-model regression in R using package lme4 (random intercepts; maximum likelihood estimation [54]). This allows accounting for the nesting of observations within both participants and cognitive enhancers which were modeled as crossed factors. Interval variables were group-centred around their mean (by cognitive enhancer [55]). Doing so allowed us to estimate the impact of our predictors on the assessments of individual cognitive enhancers while excluding variation between cognitive enhancers and participants. In the null model (Model 1, Table 2), 21% of variance lies at the level of participants (*ICC*_Participant_ in the Null model) and 37% of variance lies at the level of cognitive enhancers (*ICC*_CE_ in the Null model).

**Table 2:** Summary of the mixed-model regression with Ethical judgement as the criterion variable.

	Model 1 (null)			Model 2		
* *	*B*	*CI*	*p*	*B*	*CI*	*p*
**Fixed Parts**						
Intercept	2.97	2.35 – 3.58	< .001	2.22	1.57 – 2.86	< .001
Familiarity				0.27	0.10 – 0.44	.002
Effectiveness				0.12	0.05 – 0.19	.001
Health risk				-0.36	-0.44 – -0.27	< .001
Legal, with prescription				0.47	0.02 – 0.93	.041
Legal, sold over-the-counter				1.02	0.46 – 1.59	< .001
**Random Parts**						
Residual σ^2^	1.49			1.30		
τ_00, Participants_	0.74			0.61		
τ_00, CE_	1.24			0.86		
*N*_Participant_	148			148		
*N*_CE_	15			15		
*ICC*_Participant_	0.21			0.22		
*ICC*_CE_	0.36			0.31		
Observations	850			850		
Loglikelihood / LRT (ChiSq)	-1495.7			-1432.4 / 126.62 ***		

σ^2^ = observation variance (Level 1), τ_00, Participants_ = Participants intercept variance (Level 2), τ_00, CE_ = Cognitive enhancer intercept variance (Level 2), *N*_Participant_ = Number of participants; *N*_CE_ = Number of assessed cognitive enhancers; *ICC*_Participant_ = Intraclass correlation for participant cluster membership; *ICC*_CE_ = Intraclass correlation for cognitive enhancer cluster membership. Loglikelihood / LRT (ChiSq) = Loglikelihood and Likelihood Ratio Test.

***: *p* < .001

As can be seen in Table 2, after the inclusion of predictors (Model 2), 22% of residual variance lied at the level of participants (*ICC*_Participant_ in the Model 2) and 31% of residual variance at the level of cognitive enhancers (*ICC*_CE_ in the Model 2). The likelihood ratio test (LRT) was significant, indicating Model 2 fitted the data better than Model 1. The results confirmed all hypotheses: Familiarity with the effects of the cognitive enhancer (H1, *B* = 0.27; *p* = .002), and its perceived effectiveness for CE (H2, *B* = 0.12; *p* = .001) were positively related to positive ethical judgement. The perceived health risk of the cognitive enhancer for the user was negatively related to ethical judgement (H3, *B* = -0.36; *p* < .001). Finally, substances were more favourably assessed in terms of ethical judgement when they were perceived to be legal, compared to those perceived as being illegal (H4). This effect was stronger for substances perceived as being legal for sale over-the-counter (*B* = 1.02; *p* < .001) compared to those perceived as being legal with a medical prescription (*B* = 0.47; *p* = .041).

## Discussion

In the present study, we examined whether and how ethical judgement regarding the use of cognitive enhancers was related to the perception of four characteristics of cognitive enhancers. In line with our hypotheses, cognitive enhancers received a more favourable ethical judgment when they were perceived as more familiar, more effective, less risky for users’ health, and legal (more so if perceived as sold over-the-counter). [19] found similar results for health risk, but did not find a significant association with legal status, which may reflect the lower ecological validity of the vignette method.

Our findings suggest that increasing familiarity (in terms of knowledge) with specific cognitive enhancers (e.g., through portrayal in the media) may render ethical judgments towards their use more positive (H1), particularly if the emphasis is put on their alleged effectiveness (H2) and legality (H4) rather than on their risks for health (H3). Furthermore, higher familiarity does not appear to result in increased perceived health risks (see Table 1). Results show that 21% of the variancein ethical judgement is due to differences in participants’ average assessment of the criterion variable (*ICC*_Participant_ in the Null model). These results suggest that inter-individual differences partly explain such a variation.

It has been previously suggested that public opinion should be taken into account in designing CE-related policies because it has reached a sufficient degree of maturity on the topic [56]. Individuals who feel they know more about specific cognitive enhancers might more readily contribute to such a debate [57]. Our findings showed that higher perceived knowledge of the effects of cognitive enhancers is associated with a favourable opinion towards cognitive enhancers. Our secondary findings further suggest that such perceived knowledge is related to a reduced perception of the health risks associated with CE. It follows that public participation in policy making might lead to a distortion of the rationality of the decisions because of an overrepresentation of individuals who perceive fewer health risks attached to CE and are more favourable to CE.

The finding that more perceived knowledge is related to lower perceived health risk also suggests that when discussing CE, public health campaigns, and ideally the press, should focus first and foremost on health risks in order to maximize prevention [19, 58]. Providing information about CE might be counterproductive without an explicit focus on health risks: health literacy is a major component of public health [59]). Yet, the presentation of facts about CE in the media is generally selective and inaccurate, since there is an over-reporting of alleged increases in CE use, a mention of unrealistic benefits of CE, and an under-reporting of health risks [4]. This might indirectly result in an increased adoption of CE, as (a) individuals adapt their conduct to their altered perception of the social norm (pluralistic ignorance [60]) and (b) the ratio of expected costs and benefits is at the heart of their decisional processes [61].

### Limitations and further studies

The use of surveys assessing perceptions of cognitive enhancers that actually exist can promote ecological validity in comparison to the vignette method, but can render studies vulnerable to a variety of response biases (e.g., social desirability bias), as well as to common method variance bias [62]. Thus, although [63] has shown that concerns regarding common method variance bias have been overstated, caution in the interpretation of findings remains important when using self-report measures. We note that our study might be less subject to common method variance bias because of the use of mixed-model analyses which allow partialling out participant variance [55]. Our study employed a cross-sectional design, which limits the possibility of drawing causal inferences. Using a longitudinal design, further studies could examine whether the use of specific cognitive enhancers [64] predicts additional variance in their ethical judgement towards their use, and whether perception of health risk, effectiveness, legality and knowledge about specific cognitive enhancers predict their use.

## Conclusion

Our study suggests that individual perceived characteristics of cognitive enhancers influence people’s ethical judgement towards CE. These findings extend the incipient knowledge about the factors relating to favourable views of CE. Well-designed prevention campaign should focus on presenting the health and penal risks of cognitive enhancers, as well as the lack of evidence regarding their effectiveness.

## Appendix

A. The list of cognitive enhancers participants were requested to assess is presented below. These were randomly presented to each participant for each assessed characteristic.

Adderall

Alcohol

Ampakine

Amphetamines

Berocca *

Caffeine (e.g., coffee, tea)

Cocaine

Guanfacine

Magnesium

Methylphenidate (e.g., Ritalin)

Modafinil

Nicotine (e.g., cigarettes)

Piracetam

Tonoglutal *

Tyrosine

* Berocca and Tonoglutal are over-the-counter dietary supplements, advertised for their cognitive enhancing properties, which can be found in most drugstores in Switzerland, where the study took place

## References

[1] Smith ME, Farah MJ. Are prescription stimulants “smart pills”? The epidemiology and cognitive neuroscience of prescription stimulant use by normal healthy individuals. Psychol Bull. 2011;137: 717–41. 10.1037/a0023825 21859174 PMC3591814

[2] Dubljević V, & Ryan CJ. Cognitive enhancement with methylphenidate and modafinil: conceptual advances and societal implications. Neurosci Neuroecon. 2015;4:25–33.

[3] Franke AG, Bagusat C, Rust S, Engel A, & Lieb K. Substances used and prevalence rates of pharmacological cognitive enhancement among healthy subjects. Eur Arch Psy Clin N. 2014;264:83–90. 10.1007/s00406-014-0537-1 25214391

[4] Partridge BJ, Bell SK, Lucke JC, Yeates S, & Hall WD. Smart drugs “as common as coffee”: media hype about neuroenhancement. PloS one. 2011;6:e28416 10.1371/journal.pone.0028416 22140584 PMC3227668

[5] Illies C. The grounds of ethical judgement: new transcendental arguments in moral philosophy. Oxford: Oxford University Press; 2003.

[6] Bostrom N, & Sandberg A. Cognitive enhancement: methods, ethics, regulatory challenges. Sci Eng Ethics. 2009;15:311–341. 10.1007/s11948-009-9142-5 19543814

[7] Maslen H., Faulmüller N., & Savulescu J. Pharmacological cognitive enhancement—how neuroscientific research could advance ethical debate. Front Syst Neurosci. 2014;8:107 10.3389/fnsys.2014.00107 24999320 PMC4052735

[8] Faber N. S., Savulescu J., & Douglas T. Why is cognitive enhancement deemed unacceptable? The role of fairness, deservingness, and hollow achievements. Front Psychol. 2016;7:232 10.3389/fpsyg.2016.00232 26925027 PMC4759582

[9] Cakic V. Smart drugs for cognitive enhancement: ethical and pragmatic considerations in the era of cosmetic neurology. J Med Ethics. 2009;35:611–615. 10.1136/jme.2009.030882 19793941

[10] Forlini C, & Racine E. Autonomy and coercion in academic “cognitive enhancement” using methylphenidate: Perspectives of key stakeholders. Neuroethics-NETH. 2009;2:163–77.

[11] Glannon W. Psychopharmacological enhancement. Neuroethics-NETH. 2008;1:45–54.

[12] Bedzow I. The confused ethics of cognitive enhancers. Journal of Clinical Psychiatry and Neuroscience. 2018;1:12–14.

[13] Hogan R. Moral conduct and moral character: a psychological perspective. Psychol Bull. 1973;79: 217–32. 4699783 10.1037/h0033956

[14] Tangney JP, Stuewig J, & Mashek DJ. Moral emotions and moral behavior. Annual Rev Psychol. 2007;58:345–72. 10.1146/annurev.psych.56.091103.070145 16953797 PMC3083636

[15] Mazanov J, Dunn M, Connor J, & Fielding ML. Substance use to enhance academic performance among Australian university students. Perform Enhanc Health. 2013;2:110–8.

[16] Judson R., & Langdon SW. Illicit use of prescription stimulants among college students: prescription status, motives, theory of planned behavior, knowledge and self-diagnostic tendencies. Psychol Health Med. 2009;14:97–104. 10.1080/13548500802126723 19085316

[17] Riis J, Simmons JP, & Goodwin GP. Preferences for enhancement pharmaceuticals: The reluctance to enhance fundamental traits. J Consum Res. 2008;35:495–508.

[18] Franke AG, & Lieb K. Pharmacological neuroenhancement: substances and epidemiology In Hildt E. & Franke A. G., Cognitive Enhancement. Dordrecht: Springer; 2013 p. 17–27.

[19] Sattler S, Forlini C, Racine É, & Sauer C. Impact of contextual factors and substance characteristics on perspectives toward cognitive enhancement. PLoS One. 2013;8:e71452 10.1371/journal.pone.0071452 23940757 PMC3733969

[20] Aguinis H, & Bradley KJ. Best practice recommendations for designing and implementing experimental vignette methodology studies. Organ Res Methods. 2014;17:351–71.

[21] Hainmueller J, Hangartner D, & Yamamoto T. Validating vignette and conjoint survey experiments against real-world behavior. P Natl Acad Sci USA. 2015;112:2395–400. 10.1073/pnas.1416587112 PMC4345583 25646415

[22] Bohrn IC, Altmann U, Lubrich O, Menninghaus W, & Jacobs AM. When we like what we know–A parametric fMRI analysis of beauty and familiarity. Brain Lang. 2013;124:1–8. 10.1016/j.bandl.2012.10.003 23332807

[23] Greene J, & Haidt J. How (and where) does moral judgment work? Trends Cogn Sci. 2002;6:517–23. 12475712 10.1016/s1364-6613(02)02011-9

[24] Moll J, Zahn R, de Oliveira-Souza R, Krueger F, & Grafman J. The neural basis of human moral cognition. Nat Rev Neurosci. 2005;6:799–809. 10.1038/nrn1768 16276356

[25] Berscheid E, & Ammazzalorso H. Emotional experience in close relationships In Fletcher GJO & Clark MS, Blackwell handbook of social psychology.Vol. 2 Oxford, UK: Blackwell; 2001 p 308–30.

[26] Coupey E, Irwin JR, & Payne JW. Product category familiarity and preference construction. J Consum Res. 1998;24:459–68.

[27] Carlson JS, & Widaman KF. The effects of study abroad during college on attitudes toward other cultures. Int J Intercult Rel. 1988;12:1–17.

[28] Pettigrew TF, & Tropp LR. A meta-analytic test of intergroup contact theory. J Pers Soc Psychol. 2006;90:751–83. 10.1037/0022-3514.90.5.751 16737372

[29] Zajonc RB. Attitudinal effects of mere exposure. J Pers Soc Psychol. 1968;9:1–27. 10.1037/h0025716 5667435

[30] Partridge B, Lucke J, & Hall W. A comparison of attitudes toward cognitive enhancement and legalized doping in sport in a community sample of Australian adults. A J Bioethics. 2012;4:81–6.

[31] Smart JJC, & Williams B (1973). Utilitarianism: For and against. Cambridge: Cambridge University Press; 1973.

[32] Tobler PN, Kalis A, & Kalenscher T. The role of moral utility in decision-making: An interdisciplinary framework. Cogn Affect Behav Neurosc. 2008;8:390–401. 10.3758/CABN.8.4.390 19033237

[33] Hechter M. The role of values in rational choice theory. Ration Soc. 1994;6:318–33.

[34] Tversky A & Kahneman D. Advances in prospect theory: Cumulative representation of uncertainty. J Risk Uncertainty. 1992;5:297–323.

[35] Gibson BM, Wasserman EA, & Kamil AC. Pigeons and people select efficient routes when solving a one-way" traveling salesperson" task. J Exp Psychol Anim Behav Process. 2007;33:244–61. 10.1037/0097-7403.33.3.244 17620024

[36] Loewenstein GF, Thompson L, & Bazerman MH. Social utility and decision making in interpersonal contexts. J Pers Soc Psychol. 1989;57:426–41.

[37] Sattler S, Sauer C, Mehlkop G, & Graeff P. The rationale for consuming cognitive enhancement drugs in university students and teachers. PloS one. 2013;8:e68821 10.1371/journal.pone.0068821 23874778 PMC3714277

[38] Eisenberg AE, Baron J, & Seligman ME. Individual differences in risk aversion and anxiety. Psychol Bull. 1998;87:245–51.

[39] Zhao J, Harris M, & Vigo R (2016). Anxiety and moral judgment: The shared deontological tendency of the behavioral inhibition system and the unique utilitarian tendency of trait anxiety. Pers Indiv Differ. 2016;95:29–33.

[40] Conrad P. Wellness as virtue: Morality and the pursuit of health. Cult Med Psychiat. 1994;18:385–401. 10.1007/BF01379232 7956306

[41] Helman CG. Culture, health and illness 5th ed. London: Hodder Arnold 2007.

[42] Rozin P, & Singh L. The moralization of cigarette smoking in the United States. J Consum Psychol. 1999;8:321–37.

[43] Massey JL, & Krohn MD. A longitudinal examination of an integrated social process model of deviant behavior. Soc Forces. 1986;65:106–34.

[44] Raz J. The authority of law: essays on law and morality. Oxford: Oxford University Press; 2009.

[45] Simmonds N. Law as a moral idea. Oxford: Oxford University Press; 2007.

[46] Amonini C, & Donovan RJ. The relationship between youth's moral and legal perceptions of alcohol, tobacco and marijuana and use of these substances. Health Educ Res. 2005;21:276–86. 10.1093/her/cyh064 16234282

[47] Bilz K, & Nadler J. Law, psychology, and morality. Psychol Learn Motiv. 2009;50:101–31.

[48] Pickett JT, & Baker T. Punishment and solidarity? An experimental test of the educative-moralizing effects of legal sanctions. J Exp Criminol. 2017;13:217–40.

[49] Tyler TR. Why people obey the law. Princeton: Princeton University Press; 2006.

[50] Walker N, & Argyle M. Does the law affect moral judgments?. Brit J Criminol. 1964;4:570–81.

[51] Bergkvist L, & Rossiter JR. The predictive validity of multiple-item versus single-item measures of the same constructs. J Marketing Res. 2007;44:175–84.

[52] Fisher GG, Matthews RA, & Gibbons AM (2016). Developing and investigating the use of single-item measures in organizational research. *J Occup Health Psych*:21:3–23. 10.1037/a0039139 25894198

[53] Wanous JP, & Hudy MJ. Single-item reliability: a replication and extension. Organ Res Methods. 2001;4:361–75.

[54] Bates D, Maechler M, Bolker B, & Walker S (2014). lme4: Linear mixed-effects models using Eigen and S4. R package version;1:1–23.

[55] Snijders T, & Bosker R. Multilevel analysis: An introduction to basic and applied multilevel analysis. 2nd ed London: Sage; 2012.

[56] Fitz NS, Nadler R, Manogaran P, Chong EW, & Reiner PB. Public attitudes toward cognitive enhancement. Neuroethics-NETH. 2014;7,173:88.

[57] Jung N, Kim Y, & de Zúñiga HG. The mediating role of knowledge and efficacy in the effects of communication on political participation. Mass Commun Soc. 2011;14:407–30.

[58] Ajzen I. Nature and operation of attitudes. Annual Rev Psychol. 2001;52:27–58. 10.1146/annurev.psych.52.1.27 11148298

[59] Nutbeam D. Health literacy as a public health goal: a challenge for contemporary health education and communication strategies into the 21st century. Health Promot Int. 2000;15:259–67.

[60] Prentice DA, & Miller DT. Pluralistic ignorance and alcohol use on campus: some consequences of misperceiving the social norm. J Pers Soc Psychol. 1993;64:243–56. 8433272 10.1037//0022-3514.64.2.243

[61] Kahneman D, & Tversky A. Choices, values, and frames. Am Psychol. 1984;39:341–50.

[62] Podsakoff PM, MacKenzie SB, Lee JY, & Podsakoff NP. Common method biases in behavioral research: A critical review of the literature and recommended remedies. J Appl Psychol. 2003;88:879–903. 10.1037/0021-9010.88.5.879 14516251

[63] Spector PE. Method variance in organizational research: truth or urban legend?. Organ Res Methods. 2006;9:221–232.

[64] Pohl S., Boelsen H., & Hildt E. Moral attitudes towards pharmacological cognitive enhancement (PCE): Differences and similarities among Germans with and without PCE experience. Front Pharmacol. 2018:9:1451 10.3389/fphar.2018.01451 30618746 PMC6295456

